# Dissecting gene expression at the blood-brain barrier

**DOI:** 10.3389/fnins.2014.00355

**Published:** 2014-11-06

**Authors:** Melanie A. Huntley, Nga Bien-Ly, Richard Daneman, Ryan J. Watts

**Affiliations:** ^1^Department of Bioinformatics and Computational Biology, Genentech Inc.South San Francisco, CA, USA; ^2^Department of Neuroscience, Genentech Inc.South San Francisco, CA, USA; ^3^Department of Pharmacology, University of California, San DiegoLa Jolla, CA, USA

**Keywords:** blood-brain barrier, transcriptome, genomics, brain endothelial cells, expression profiling

## Abstract

The availability of genome-wide expression data for the blood-brain barrier is an invaluable resource that has recently enabled the discovery of several genes and pathways involved in the development and maintenance of the blood-brain barrier, particularly in rodent models. The broad distribution of published data sets represents a viable starting point for the molecular dissection of the blood-brain barrier and will further direct the discovery of novel mechanisms of blood-brain barrier formation and function. Technical advances in purifying brain endothelial cells, the key cell that forms the critical barrier, have allowed for greater specificity in gene expression comparisons with other central nervous system cell types, and more systematic characterizations of the molecular composition of the blood-brain barrier. Nevertheless, our understanding of how the blood-brain barrier changes during aging and disease is underrepresented. Blood-brain barrier data sets from a wider range of experimental paradigms and species, including invertebrates and primates, would be invaluable for investigating the function and evolution of the blood-brain barrier. Newer technologies in gene expression profiling, such as RNA-sequencing, now allow for finer resolution of transcriptomic changes, including isoform specificity and RNA-editing. As our field continues to utilize more advanced expression profiling in its ongoing efforts to elucidate the blood-brain barrier, including in disease and drug delivery, we will continue to see rapid advances in our understanding of the molecular mediators of barrier biology. We predict that the recently published data sets, combined with forthcoming genomic and proteomic blood-brain barrier data sets, will continue to fuel the molecular genetic revolution of blood-brain barrier biology.

## 1. Historical perspective on datasets

In 1967 using exogenous peroxidase, Reese and Karnovsky demonstrated the subcellular localization of the blood-brain barrier (BBB) to be the endothelium forming the walls of vessels in the brain (Reese and Karnovsky, 1967). Two structural characteristics distinguished these endothelial cells from those in the heart and skeletal muscle: the presence of tight junctions, and the paucity of micropinocytotic vesicles. Early studies used microscopy and immunohistochemistry to visualize the presence and localization of a handful of known proteins within brain endothelial cells (BECs) (Risau et al., 1986), while *in situ* hybridization filled in the gaps for questions about expression patterns when antibodies were not available for specific targets. Nearly fifty years later, the underlying genes and developmental cascades that result in the unique traits of BECs are just beginning to be understood. Current critical advancements in our ability to both purify and culture BECs now allow for the study of their molecular mechanisms at an unprecedented level of breadth and depth.

Since the seminal discovery that brain endothelial cells limit the diffusion of blood-borne molecules, the following decades of work have made it clear that the BBB is not just a physical wall but a complex regulated physiology that allows the BECs to determine the movement of ions, molecules and cells between the blood and the neural tissue. At the heart of this physiology are molecular and structural specializations of BECs that differentiate them from endothelial cells in non-neural tissue. BECs are held together by tight junctions, which form a tight paracellular barrier that polarizes the cells into distinct luminal and abluminal membrane compartments. This tight paracellular junction coupled with the extremely low levels of transcytosis creates a physical barrier to hydrophilic molecules and allows cellular transport properties to determine movement of molecules between the blood and the brain. To regulate this movement, BECs express a series of different transporters that generally fall into two categories: efflux transporters and nutrient transporters (for an in depth review of BBB transporters see Saunders et al., 2013). Briefly, efflux transporters, such as ABCB1 (also known as p-glycoprotein, or Pgp), face the luminal surface and use energy generated from the hydrolysis of ATP to pump a wide array of lipophilic substrates up their concentrations gradients into the blood, thus limiting the passive diffusion of hydrophobic molecules into the brain. Nutrient transporters, such as Glut-1 (glucose transporter SLC2A1), Lat-1 (amino acid transporter light chain, SLC7A5) and Mct-1 (monocarboxylate transporter, SLC16A1), facilitate the movement of specific substrates down their concentration gradients, allowing specific molecules into or out of the brain. Thus, the BBB is not solely a physical barrier but it is a series of complex regulated physiological specializations of the BECs that control the microenvironment of the brain. Furthermore, work has demonstrated that many of these specializations of the BECs are not intrinsic, but regulated by interactions with neural cells including CNS pericytes and astrocytes, and immune cells including microglia and macrophages (Hudson et al., 2005; Armulik et al., 2011). Identifying the molecular signature of BECs and how they differ from ECs in other tissues, as well as key cell-cell signaling interactions in the neurovascular unit, are questions at the forefront in understanding the physiology of the BBB.

## 2. The utility of datasets as resources

High quality gene expression data has significantly impacted the direction of investigation by allowing for a better molecular understanding of BBB development, function, and dysfunction. Well-powered gene expression studies can validate hypothesis driven queries or open previously unexplored avenues of research in unbiased directions. Models of acute BBB dysfunction, such as stroke or ischemia, aid in elucidating mechanisms of impairment for development of therapeutics or preventative treatments. An understanding of BBB-specific transporter, receptor, and ion channel expression levels could benefit drug development efforts by aiding in the design of selective compounds for those that could evade efflux pumps (Pottiez et al., 2009) or utilize receptors to enhance therapeutic uptake in brain (Yu and Watts, 2013).

The variety of questions and issues that can be addressed related to BBB development and disease have vastly improved, thanks in part to genetic engineering approaches in rodents and other species, improved cellular isolation techniques, and the availability of bioinformatics software and experts that facilitate the mining of data sets for relevant genes worthy of further investigation. The combination of “big data” sets and molecular-genetic tools is beginning to elucidate biological mechanisms in many arenas, and our understanding of BBB biology is benefiting from this revolution.

### 2.1. Key parameters for BBB genomic studies

**Figure 1:**
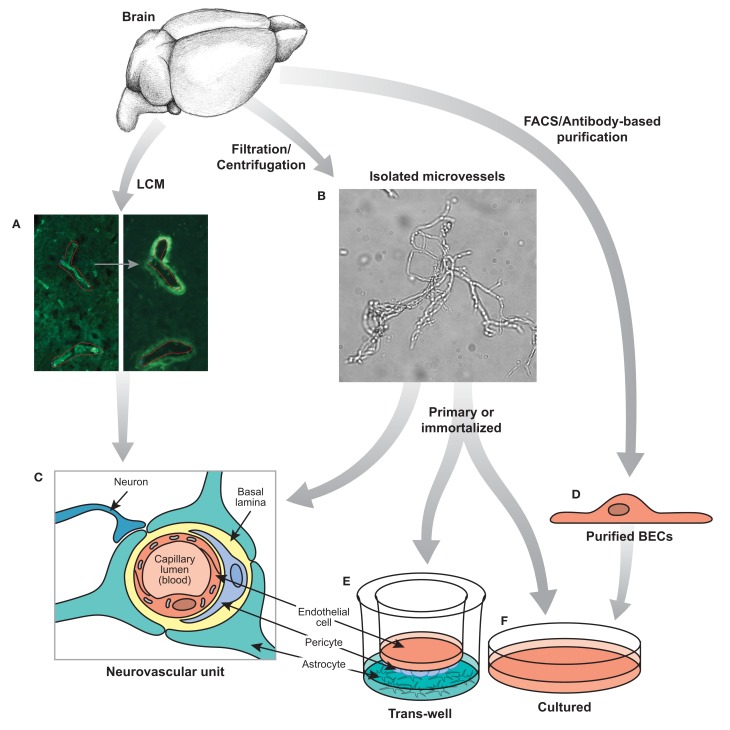
**Techniques for isolating BECs**. **(A)** Laser capture microdissection or **(B)** mechanical filtration of brain microvessels can be used to isolate segments of the neurovascular unit **(C)**. FACS or other antibody based purification **(D)** can be performed to isolate BECs acutely. Isolated microvessels **(B)** and purified BECs **(D)** can be further cultured in a trans-well system **(E)** or other relevant *in vitro* culture systems **(F)**. Experiments based on the neurovascular unit **(C)**, purified primary BECs **(D)** and cultured BECs **(E,F)** will each provide valuable, but different insights into the biology of the BBB.

The cellular complexity of the BBB presents a significant obstacle in the gathering of meaningful gene expression data. Brain endothelial cells are surrounded by the mural cells–pericytes and smooth muscle cells–and are further encapsulated by a basement membrane (see Figure 1C). The ensheathment of the vessels by astrocytic endfeet, likely receiving signals from neurons and microglia, serve to further regulate brain vascular function (Abbott, 2005). Therefore, these various cell types are intimately connected and unite in maintaining the integrity of the BBB. Thus a global analysis of BBB gene expression may necessitate the co-purification and co-analysis of these important non-BEC cell types along with BECs (Shusta, 2005). Early efforts to harvest brain vasculature for genomic analysis captured a majority of the cell types associated with BECs of the neurovascular unit. These earlier protocols relied on mechanical brain dissociation to isolate microvascular components, a technique known as vascular enrichment (Triguero et al., 1990; Boado and Pardridge, 1991), which uses a series of filtrations through nylon membranes or with glass beads followed with gradient centrifugation steps (Betz et al., 1979; Yousif et al., 2007). This contrasted with laser capture microdissection (LCM) methods for single capillary fragment isolation, which allows for highly localized sampling from specific human and mouse brain sections and could be restricted to diseased regions (Ball et al., 2002; Mojsilovic-Petrovic et al., 2004). Again, both methods do not specifically yield pure populations of BECs specifically, however, they do allow for a sampling of cells associated with the neurovascular unit and generally enrich for BECs for inferences to be drawn about CNS microvasculature.

Thus, several important considerations must be taken into account when designing genomic experiments to analyze the BBB. The most important parameter is to determine the desired cells or tissues to be analyzed and the method of purification. Because the key properties of the BBB are specializations of BECs, many studies have used methods to purify ECs from brain tissue, whereas others have used crude approaches to purify vessels (endothelial cells and pericytes) or entire fragments of the neurovascular unit (see Figure 1). In addition, the vasculature of the brain contains many different segments of the vascular tree including arteries, arterioles, capillaries, post-capillary venules, venules, and veins (Dorr et al., 2007), all of which contain barrier properties but each segment may have different transport, metabolic, signaling and cell adhesion properties. Gradient centrifugation has been used to specifically isolate microvessels (capillaries and post-capillary venules), whereas purification based on markers of ECs will contain BECs from all segments of the vascular tree. Also taken into consideration should be the species from which the samples are isolated, whether human, rodent or other model organism, and whether to examine acutely purified or cultured cells. The second parameter is the method of analysis. For transcriptomic generation of EST libraries, subtractive hybridization, microarrays and RNA sequencing are all common methods to examine RNA. Different methods of RNA extraction, including whole RNA, mRNA, small RNA, and tagged-ribosome isolation can highlight specific RNAs of interest. In addition, proteomics, metabolomics, lipidomics, epigenomics, and other “omics” analysis can give an even broader overview of the molecular composition of the BBB. The third parameter is the effective comparisons used for differential gene expression analysis. Identifying all the transcripts in a given cell can be challenging to decipher, however, generating a comparison of two cells that vary only for a given function can greatly narrow down the search for important genes. In the case of the BBB, several important comparisons have been made, including comparing BECs with whole brain extracts to identify EC specific genes, as well as comparing BECs with peripheral ECs to identify BBB-specific specializations (see Table 1 for a summary of recent BBB expression profiling studies). The fourth important parameter to determine is the physiological setting in which to analyze the BBB. Much work has been done to identify the gene expression signature of the BBB in a healthy adult animal, but studies have also examined the BBB during development, in different neurological disease settings, in mice with different mutations that affect BBB formation and function, or with the addition of different pharmacological agents. Each of these parameters will greatly influence the data set obtained and therefore careful consideration must be taken to ensure the best method of BEC purification and analysis.

**Table 1:** **Recent genomewide microarray expression data sets for the BBB**.

**Method of BBB isolation**	**Relevant study details**	**Species**	^*^Dev study	**Other tissues**	**Disease**
**ANTIBODY-BASED/FACS PURIFICATION**					
Ben-Zvi et al., 2014	Tie2-GFP embryonic ECs from brain and lung	Mouse		+	
Tam et al., 2012	ECs from liver, lung, brain in embryo, pup, adult	Mouse	+	+	
Daneman et al., 2010a	Adult VCs from liver, lung, brain (+ postnatal), BECs and parenchyma	Mouse	+		
Daneman et al., 2010b	BECs from PDGFRb KO mice; BECs and pericytes from wild-type	Mouse	+		
Daneman et al., 2009	Tie2-GFP adult ECs from brain, liver and lung	Mouse		+	
Lyck et al., 2009	Primary PECAM+ BMECs, cultured b.End5 cells and BMECs	Mouse			
**LCM**					
Dieterich et al., 2012	Blood vessels associated with gliobastoma tumors	Human			+
Cunnea et al., 2010	Brain microvessels in multiple sclerosis patients	Human			+
Giger et al., 2010	Pyramidal neurons and vascular endothelium	Human			
Harris et al., 2008	Neurons and microvessels in schizophrenia patients	Human		+	+
**FILTRATION/CENTRIFUGATION**					
Wang et al., 2012a	BMVs from Alzheimer's patients and controls	Human			+
Armulik et al., 2010	BMVs from wild type, Tie2Cre, R26P+/0, pdgfb-/- (pericyte deficient)	Mouse	+		
He et al., 2011	BMVs from adult male rats	Rat			
**CELL CULTURE**					
Reijerkerk et al., 2013	Naive hCMEC/D3 cells with TNFα, IFNγ; astrocyte-media cultured	Human			
Lopez-Ramirez et al., 2013	hCMEC/D3 cells with TNFα and IFNγ	Human			+
Urich et al., 2012	hCMEC/D3 cells and human primary BECs	Human			
Barbier et al., 2011	HBECs with platelets ± TNF, plasmodium infected RBCs	Human			+
Himburg et al., 2010	BEC cultures and non-brain EC cultures	Human		+	
Li et al., 2008	HBMECs treated with amyloid-β	Human			+

* *Developmental stages. BEC, brain endothelial cell; BMEC, brain microvascular endothelial cell; BMV, brain microvessel; HBEC, human brain endothelial cell; HBMEC, human brain microvessel endothelial cells; EC, endothelial cell; RBC, red blood cell; VC, vascular cell*.

### 2.2. Methods of analysis

Earlier transcriptomic approaches that yielded important initial insights on brain microvascular specific expression data included suppression subtractive hybridization and serial analysis of gene expression (SAGE) (Li et al., 2001; Shusta et al., 2002; Enerson and Drewes, 2006; Calabria and Shusta, 2008). For a detailed review on these techniques and early results, see Shusta (2005), Pottiez et al. (2009). A distinguishing aspect of the SAGE study of rat brain microvessels was the comparison to rat neocortex and hippocampus tag catalogs and the resulting identification of 864 BBB enriched genes.

Today, expression microarrays offer a mature and commonly used platform to differentially compare gene expression between two samples. One of the earliest BBB gene expression studies using microarray established a protocol to profile human brain endothelial cells co-cultured with astrocytes under dynamic conditions with controlled intraluminal flow (Marroni et al., 2003). The authors observed that exposure to increased flow up-regulated cytoskeletal genes while also inhibiting cell cycling genes.

In the era of microarray data sets on the BBB (see Table 1), the number of genes that are queried on commercially available chips has increased several orders of magnitude, moving the field into truly high-throughput experiments and data. In addition, relatively small amounts of cells or tissue are now required for genome-wide transcriptomic approaches, thus facilitating a wide range of studies. Advancements and maturity of computational tools for this type of data analysis are now available allowing investigators to dive into these data sets and identify meaningful connections (Irizarry et al., 2003; Gentleman et al., 2004; Smyth, 2004; Falcon and Gentleman, 2007; Kauffmann et al., 2009). Online repositories, such as Gene Expression Omnibus and the European Nucleotide Archive, now allow researchers to deposit genome wide expression data sets to be queried and re-used by other groups. Indeed, many of the studies listed in Table 1 have made use of previously published expression data sets from the BBB as an integral part of their analyses. The importance of continuing to expand the public availability of such data sets as an ongoing resource for the community cannot be overstated.

A discussion of newer techniques, such as next generation RNA-sequencing, will follow at the conclusion of this review.

## 3. Experimental paradigms analyzed

Figure 1 summarizes the most common methods for isolation and purification used in BBB genome-wide expression studies. As many recent studies have shown, *in vitro* models of the BBB do not fully recapitulate the complexity of an intact organism. The use of newer techniques such as fluorescence activated cell sorting (FACS), immunopanning, magnetic bead immunoprecipitation, as well as advances to LCM may aid in acutely harvesting specifically BECs, or an enriched population of BECs, for downstream transcriptomic and proteomic studies. Navone et al. (2013) document recent protocols for the isolation of human and mouse brain microvascular endothelial cells. However, no technique or protocol is perfect as there are subtle considerations to be aware of which may factor largely into downstream data analysis. Even careful acute isolation and genomic profiling of BECs may be susceptible to general expression changes induced by mechanical shearing of brain parenchyma and/or microvessel tissue, enzymatic digestion, temperature fluctuations, etc. Careful pair-wise comparisons with the proper controls undergoing similar handling are critical to minimize bias in the data.

### 3.1. Acute isolation and purification of brain microvascular cells

The use of antibody-based techniques to isolate rodent brain endothelial cells has been reported in several recent studies. Magnetic beads coupled to murine or rat antibodies against CD31/PECAM allow for immunoprecipitation of endothelial cells from homogenates of brain (Lyck et al., 2009) and possibly other vascular organs for direct comparisons. Likewise, antibodies against CD31 can be immobilized onto culture plates for immunopanning enrichment of brain homogenates for acute isolation of BECs (Zhou et al., 2014a) or the use of other cell surface receptors such as anti-PDGFRβ for pericytes (Daneman et al., 2010b). This approach also allows for a negative selection step against microglia or pericytes to remove these non-BEC subtypes from the overall homogenate. A third BEC-specific antibody or antigen-selection technique utilizes fluorescence-activated cell sorting which would use either a fluorophore-coupled anti-BEC cellular marker (Daneman et al., 2010b; Tam et al., 2012) or transgenic mice that harbor BEC-specific promoters to direct expression of a fluorescent protein, such as Tie2-GFP mice (Daneman et al., 2009, 2010a; Ben-Zvi et al., 2014).

Moreover, LCM has experienced many improvements including the addition of immunostaining protocols and brighter, more specific dyes for identification of brain endothelia (Macdonald et al., 2008). Recent immuno-LCM studies have described differences in gene expression of brain capillaries vs. venules, and highlighted important roles for their heterogeneity (Macdonald et al., 2010). Increased antibody availability for other neurovascular unit cell types have also enabled cross comparison of gene expression as well as validation of cell type specificity of the data sets generated by mass spectrometry (Murugesan et al., 2011). Advances in equipment such as microscopes with automated stages, higher quality cameras, and sophisticated software have enabled more consistent tissue sampling and higher throughput.

### 3.2. *In vitro* BBB models

Immortalized cell lines derived from rodent or human brain endothelial cells have been frequently used as a model system for gene expression studies despite their questionable physiological relevance. Cultured cells, especially those that can be passaged indefinitely, represent a key model system that allows for a high level of cellular homogeneity, consistency, and experimental reproducibility. Key components of the neurovascular unit can be reconstituted *in vitro* by growing BECs on filter inserts that sit above pericytes and/or astrocytes, allowing signaling mechanisms between these cell types to remain largely intact. Conversely, *in vitro* models may still lack the complex interactions between these cell types and the endogenous extracellular matrix, as well as surrounding neurons and microglia that exist *in vivo*. Complicated disease processes that are also in play *in vivo* are likely difficult to fully model in a transwell culture system and suffer from numerous drawbacks (Hawkins and Egleton, 2008; Pottiez et al., 2009). In other words, the neurovascular unit is essentially an organ system with numerous cellular interactions that make it virtually impossible to replicate outside of the organism. However, these culture paradigms also do enable investigators to determine what individual cell types can do in the absence of other cells, or with defined cellular interactions. For instance, culturing BECs alone can give an idea of which BBB properties are independent of signaling interactions within the neurovascular niche. Furthermore, by adding feeder layers of individual cells, such as astrocytes or pericytes, or signaling molecules or pharmacological agents, *in vitro* models can aid in determining how these factors directly influence BEC gene expression.

Primary BEC culture models obtained from bovine or rodent acutely isolated brain microvessels present another system for directed studies on BBB maintenance and function. Early efforts relied on isolation and enrichment of brain capillaries from rats followed by enzymatic digestion of the basement membrane to allow migration of endothelial cells for growth on an artificial substrate (Bowman et al., 1981). Bovine models have allowed for several benefits including greater amounts of starting material obtained from one animal, less variability between cultures from the same starting material, and importantly, retention of endothelial characteristics after serial passaging (Goetz et al., 1985).

The establishment of human cerebral microvessel cultures also yielded early, important insights into BBB function (Vinters et al., 1987). These culture systems were improved upon as tighter barriers were introduced with co-culture models containing astrocytes and by introducing intraluminal flow (Marroni et al., 2003). Human brain microvessel endothelial cells (HBMEC) have also been treated with amyloid-β to determine their gene expression changes using a cDNA array and identified a putative protective role for STC1 (Li et al., 2008).

### 3.3. Comparisons of acute vs. cultured BBB models

Whether *in vitro* BBB models were derived from immortalized cell lines or acutely isolated from mouse or human brains, extensive efforts to characterize their gene expression signature have repeatedly revealed their limited approximation of *in vivo* complexity. Comparisons of BBB gene expression profiles from acutely isolated mouse brain endothelial cells vs. a period of brief culturing, or compared against a commonly used mouse brain endothelioma line (bEND5) revealed evidence of dedifferentiation of BEC qualities (i.e., reduced levels of tight junction proteins and amino acid transporters) induced by primary culture or immortalization (Lyck et al., 2009). A similar study which used suppression subtractive hybridization revealed that some transport-related brain endothelial genes were subsequently downregulated after BECs spent several days in culture compared to freshly isolated rat brain microvessels (Calabria and Shusta, 2008). The same study compared cultures containing puromycin (which decreases numbers of contaminating pericytes) for BEC expression and also the effect of hydrocortisone on cultures. More recently, transcriptome data from immortalized human BEC line hCMEC/D3 and primary human BEC (Urich et al., 2012) were compared with published data on acutely isolated mouse BECs (Daneman et al., 2010a) to highlight differences in levels of endothelial marker expression. The authors found that although the human cells demonstrated expression of tight junction proteins, transporters and receptors, their levels were dramatically reduced compared to acutely isolated mouse BECs (Urich et al., 2012).

Nevertheless, using *in vitro* BBB models may yield some important initial insights into BEC response to external stimuli or stress. hCMEC/D3 cells have been recently profiled for their transcriptional response to TNFα and IFNγ and gene expression signatures identified span from factors involved in antigen presentation, cellular adhesion, cytokine-induced signaling pathways, to reduced transporter expression (Lopez-Ramirez et al., 2013). These data were compared to similar historical data sets that included TNFα-treated human umbilical vein (HUVEC) and primary human cerebral endothelial cell cells (Franzen et al., 2003).

### 3.4. Models of BBB development

Wnt/β-catenin signaling has been implicated in driving CNS specific angiogenesis and regulating BBB formation and maintenance (Liebner et al., 2008; Stenman et al., 2008; Daneman et al., 2009; Wang et al., 2012b; Zhou et al., 2014b). Using microarray expression profiling comparing BECs with ECs from non-neural tissue, Daneman and colleagues identified that genes downstream of Wnt/β-catenin signaling were greatly enriched in endothelial cells of the brain but not liver or lung (Daneman et al., 2009, 2010a). Performing sophisticated genetic and functional experiments, the authors demonstrate that Wnt/β-catenin signaling is required to drive CNS-specific angiogenesis but not angiogenesis in other tissues. To identify if this CNS-specific angiogeneic program also induced BBB-specific gene expression in the endothelial cells, the authors utilized gene expression profiling of purified mouse brain endothelial cells cultured with and without Wnt ligands to find that Wnt induced the expression of BBB-specific nutrient transporters in the endothelial cells, and further showed that loss of Wnt signaling *in vivo* led to loss of Glut-1 expression in endothelial cells. More recently, Zhou et al. (2014b) demonstrated nearly identical canonical Wnt signaling mechanisms of development and maintenance between both the blood-brain and blood-retinal barriers.

The characterization of molecular cues and signaling pathways that guide both physiological and pathophysiological CNS angiogenesis may also stand to benefit greatly from expanded efforts at expression profiling of BECs within various parts of the neurovascular unit. Common guidance molecules that regulate both nerve and vascular remodeling such as Sema/Plex/Nrp, Slit/Robo, Netrin/Unc5, VEGF, and its tyrosine kinase receptors Flt-1 and Flk-1, have been extensively studied, however, the role of the canonical axon guidance molecules in CNS angiogenesis is less studied (Tam and Watts, 2010). Transcriptomics may also enable future studies on understanding the role of TGFb or EphB receptors and ligands in determining arteriovenous identity within the brain vasculature (Adams and Alitalo, 2007; Engelhardt and Liebner, 2014).

In addition to the involvement of Wnt signaling in BBB angiogenesis and barriergenesis, the role of growth factors and kinases beyond VEGF and Notch have also been further characterized. Mice deficient in PDGF-B lack microvascular pericytes and succumb to lethal microaneurisms during development due to incomplete brain vascularization (Lindahl et al., 1997). More recent studies on the importance of pericytes in barriergenesis and maintenance reported that significantly reduced pericyte coverage in PDGFRβ null fetal mice translated into increased permeability (Daneman et al., 2010b). Transcriptome signatures of BECs isolated from null mice were compared to wild type mice, which revealed increases in Angpt2, a Tie2 ligand involved in vascular permeability of peripheral endothelial cells but no downregulation of BBB-enriched genes. Thus pericytes may regulate the BBB by inhibiting the expression of genes that normally make endothelial cells leaky. In addition, the expression profile of pericytes acutely isolated from wild type mice was also generated, further providing a valuable data set for BBB neurovascular unit transcript comparisons (Daneman et al., 2010b).

For an in depth review on recent studies of developmental barriergenesis and repair, see Obermeier et al. (2013), Siegenthaler et al. (2013), Engelhardt and Liebner (2014).

### 3.5. Evolutionary studies of the BBB

Two distinct cellular mechanisms of BECs appear to be evolutionarily conserved across all vertebrates and some invertebrates, including the fruit fly model organism, *Drosophila melanogaster*. These conserved traits include the tight junctions that prevent paracellular diffusion, and ATP binding cassette (ABC) transporters that efflux molecules back into the vascular luminal space. The *Drosophila* genome encodes proteins which are nearly identical in sequence to those that comprise vertebrate tight junctions (Wu and Beitel, 2004; Banerjee et al., 2006), and whose disruption leads to defects in humoral-CNS barrier function (Schwabe et al., 2005; Stork et al., 2008). Further, Mayer et al. (2009) established that the fly protein Mdr65 functions as an ABC transporter in the *Drosophila* hemolymph-brain barrier.

Validation of gene expression data in other species with a simplistic BBB may expedite the search for candidate genes that warrant further study in rodents or human tissue. For example, we recently profiled the rodent BBB at three developmental stages, used zebrafish to confirm the necessity of candidate genes in regulating angiogenesis, and followed with subsequent analysis of genetic interactions of two death receptors, DR6 and TROY, by returning to mice to confirm a role for these genes in rodent BBB development (Tam et al., 2012). As such, conserved pathways can be discovered in one species and further characterized in more tractable models. In fact, fruit fly, grasshopper, and zebrafish are gaining popularity as models for high throughput screening of BBB drug permeability studies (Nielsen et al., 2011; Geldenhuys et al., 2012) It is this underlying premise of evolutionary conservation which provides confidence that understanding the BBB in rodent and other model organisms will be relevant to the human BBB.

Primate studies, on the other hand, can illuminate more recent evolutionary innovations relevant to the human BBB. Gene expression differences between neurons and BECs from human postmortem tissue isolated by LCM were compared with published data sets on chimpanzees and rhesus macaques for analysis of rates of transcriptome evolution in neuronal vs. endothelial cell types (Giger et al., 2010). The authors found that transcriptomes of neurons and endothelial cells evolve at different rates within brain tissue. This finding adds complexity to the challenge of translating BBB results from other species, even closely related species, into human models.

### 3.6. Human BBB data sets focused on aging, disease, and dysfunction

During normal aging, the blood brain barrier experiences functional changes, which can result in impairment and dysfunction of the barrier. Marques et al. (2013) provide a review of these changes in both the BBB and blood-CSF barriers. In the BBB, these changes include loosening of tight junctions, increases in pinocytotic vesicles, and decreases in mitochondrial density within ECs. The authors highlight the similarity between normal aging, and CNS disease related changes of the BBB. Since human BBB data from patients typically comes from those with obviously progressed symptoms, it is a challenge to determine if the changes in BBB function are causal or consequential to disease progression. Improvements in animal models of CNS diseases with BBB impairment may help answer such questions.

Nevertheless, several human studies in diverse disease paradigms have sought to understand vascular expression differences between healthy control and disease tissue. Microvascular endothelial cells from postmortem schizophrenia brains were isolated by LCM and their functional profile was compared to neurons and control tissue in a study by Harris et al. (2008). They found that the cerebral microvasculature of schizophrenic patients displayed a hypo-inflammatory status and this observation was reproducible on a separate microarray platform. This gene set could be useful in comparison studies with similar rodent models of cognitive dysfunction or with human BBB gene sets from patients with other cognitive or behavioral impairments. Likewise, the use of LCM to isolate vessels from glioblastoma multiforme brain tissue followed by microarray analysis and qPCR validation identified uniquely upregulated genes specific to tumor tissue and absent in nonmalignant human brain (Pen et al., 2007). Interestingly, a similar study from another group did not identify the same upregulated genes and describe a different expression signature (Dieterich et al., 2012). Another recent study also used LCM to query genes expressed at the BBB from multiple sclerosis lesions isolated from postmortem tissue (Cunnea et al., 2010).

Brain endothelial cell transcriptomes from human neurodegenerative diseases have been distinctly underrepresented. However, there is no shortage of studies reporting genome-wide data sets from Alzheimers disease brain or sub-regions such as hippocampus (Blalock et al., 2004; Dunckley et al., 2006; Webster et al., 2009; Blalock et al., 2011). To the best of our knowledge, the only published data set generated thus far representing the BBB in human neurodegeneration is from a study where brain microvessels from control and Alzheimers disease tissue were transcriptionally profiled to identify abnormal gene expression or differentially expressed genes (Wang et al., 2012a). A comparison of this data set with brain microvasculature gene expression data derived from mouse models of Alzheimer's disease may serve as a useful tool for model validation.

### 3.7. Rodent BBB data sets in models of neurodegeneration and disease

Transgenic mouse models of Alzheimers disease have been instrumental to the study of amyloid-β accumulation and its role in neuronal impairment and cognitive dysfunction. Transcriptome analysis of whole brain, specific subregions, various disease time points, and in multiple Alzheimer's disease transgenic models or after experimental treatments has been performed (Dickey et al., 2003; Reddy et al., 2004; Lazarov et al., 2005; Gatta et al., 2014). Genomic studies specifically using mouse brain vasculature from Alzheimer's disease models, on the other hand, remain sparse. The most recent study used a proteomics approach from homozygous Tg-SwDI mice which harbor three familial, autosomal dominant mutations on APP, and demonstrate sustained increases in vascular amyloid-β accumulation over time (Searcy et al., 2014). The authors compared brain microvessel fractions from 3- and 9-month old wild type and transgenic mice for aging-related effects on the mouse vascular proteome as well as amyloid-β-induced effects. They found distinct sets of proteins changed in young and old wild type and transgenic mice, indicating a differential effect of age on the cerebrovasculature.

An experimental mouse model of multiple sclerosis, experimental autoimmune encephalomyelitis, is characterized primarily by dysfunction of the BBB. Functional studies have produced insights into the role of particular genes, such as the chemokine CXCL12, and the permeability of the BBB in experimental autoimmune encephalomyelitis (McCandless et al., 2006, 2009). However, to our knowledge, there does not yet exist a genome wide expression data set derived from the BBB in this important model of multiple sclerosis.

Metabolic diseases, with obesity a leading factor, may also have a multitude of effects on the BBB and as a result, CNS function. A recent study on diet-induced obesity using a mouse model profiled the proteomic changes in brain vasculature after 2 months of high fat diet chow administered starting at 2 months of age (Ouyang et al., 2014). The samples were compared directly with mice maintained on normal chow and the authors found 47 downregulated and 2 upregulated proteins, indicating that in general, diet-induced obesity suppressed metabolic activity in brain microvessels (Ouyang et al., 2014).

Rodent models exist for other neurological diseases associated with BBB dysfunction, including stroke, epilepsy, ALS, and neuromyelitis optica. However, to date, there is a void of genome wide expression data on brain endothelial cells from these important models. Future data sets derived from these models will be invaluable in bridging the knowledge gap between our understanding of healthy BBB maintenance and BBB dysfunction in disease.

Another genomic data set that is publicly available and could be used as a valuable cross-reference tool describes the transcriptional changes in four rat brain tissues/regions including striatum, and parietal cortex, choroid plexus, and the meninges and its associated vasculature (Bowyer et al., 2013). This study also included a time course analysis to profile the effects of amphetamines or environment-induced hypothermia on choroid plexus and the meninges.

## 4. Summary of insights from BBB genomic studies

### 4.1. Overview

Genomic studies on the BBB have provided a number of large data sets identifying genes that are expressed by BECs, enriched in BECs compared to peripheral ECs, and also genes that change levels during development or disease context. The challenge now is to sort through these vast data sets and untangle which of these large numbers of genes are functionally important for the formation and function of the BBB, and which can be targeted to either modulate the BBB during neurological disease or to use as carriers for drug delivery across the BBB. Two positive outcomes have arisen from these data sets: (1) many of these analyses that use completely different methodologies have identified the same BBB genes, and (2) virtually all of the data sets have verified the expression and BBB-enrichment of commonly studied genes important for BBB function including tight junctions (claudin 5, occludin, Zo-1, Zo-2), efflux transport (P-glycoprotein, and ABCG2, also known as Bcrp), nutrient transport (Glut-1, Mct-1, Lat-1) and others (basigin, carbonic anhydrase 4). The strong reproducibility and ability to verify positive hits suggests that these data sets will indeed provide an extremely strong blueprint for identification of novel genes important for BBB function.

### 4.2. Signaling regulators

Several important discoveries have already been generated from these data sets. For instance, the identification that a cluster of Wnt/β-catenin signaling components and downstream targets (Fzd6, Lef1, Axin2, Dixdc1, Apcdd1, Troy) are greatly enriched in BECs compared to peripheral ECs led to the hypothesis that Wnt may be an important regulator of the BBB. As discussed above, several groups have verified and identified that Wnt/β-catenin signaling is required for driving CNS-specific angiogenesis, BBB induction and maintenance (Liebner et al., 2008; Stenman et al., 2008; Daneman et al., 2009; Wang et al., 2012b). Beyond the morphogens Wnt and Shh, and the canonical VEGF and Notch signaling pathways, a further understanding of other growth factors and kinases involved in angiogenesis have also been possible from expression data. Moreover, gene cluster/pathway analysis can provide an important method to sift through large data sets to find groups of genes that are functionally important. Other clusters associated with BBB-enrichment are genes involved in retinoid signaling, amino acid metabolism and glycolysis, suggesting that these may be important for unique function of BECs.

### 4.3. Molecular components of the barrier

Many groups have used these data sets to identify individual genes that are important for BBB function. Lipolysis stimulated receptor (Lsr) was identified as enriched in BECs compared to peripheral ECs (Daneman et al., 2010a), has recently been shown to recruit tricellulin/Marveld2 to tricellular tight junctions for the formation of epithelial barriers (Masuda et al., 2011), and could potentially have a similar function at the BBB. Like Lsr, Marveld2 was identified as enriched at the BBB along with other tight junction components such as Jam4, Mpp7 and Cgnl1 (Daneman et al., 2010a), suggesting that these may also be important for formation of BBB tight junctions. Interestingly, the genomic data sets have not added much clarity as to which claudins may be important for BBB function. While it is well established that claudin 5 is most critical in transmembrane adhesion for BEC tight junctions (Nitta et al., 2003), various studies have also implicated claudin 1, claudin 3, claudin 12 and other claudins as expressed by BECs (Liebner et al., 2000; Nitta et al., 2003; Wolburg et al., 2003; Krause et al., 2008; Pfeiffer et al., 2011). Several different genomic data sets have essentially come to different conclusions as to which claudins are expressed by BECs. Array studies on purified mouse BECs have suggested that in addition to claudin 5, claudin 12 is the other claudin expressed by BECs (Daneman et al., 2010a; Tam et al., 2012), while cultured cells have identified claudin 3 as a component of BEC tight junctions (Liebner et al., 2008), and proteomics on human passaged endothelial cells have identified a wider array of claudins present at these junctions (Ohtsuki et al., 2013). These differences may result from species differences, differences in acutely isolated vs. cultured cells, or different sensitivities of analyses, and further work needs to be done to determine which are the important molecules.

### 4.4. Transporters and drug delivery targets

The genomic data sets have also identified a huge number of molecular transporters highly expressed and enriched in BECs compared to peripheral ECs. In addition to widely studied BBB transporters (Glut-1, Lat-1, Mct-1), a number of other transporters have been identified and include molecules that transport neurotransmitters (Slc6a6, Slc6a17), thiamine (Slc19a3), nucleotides (Slc25a33), carnitine (Slc25a20), zinc (Slc20a1, Slc39a10) and riboflavin (Slc52a2/Gpr172b) (Lyck et al., 2009; Daneman et al., 2010a). Determining the expression and function of this wide array of transporters may be important to understand the nutrient requirements of the CNS. A recent study by Ben-Zvi et al. (2014) selectively profiled E15.5 embryos for transcripts enriched at the BBB vs. lung endothelium and identified Mfsd2a, a lipid transporter required for docosahexaenoic acid transport (Nguyen et al., 2014), further confirming its high expression levels. Analyzing the expression of transporters and signaling components may also identify novel molecules to target for drug delivery to the CNS. The most commonly utilized target for BBB-specific receptor-mediated drug delivery to the CNS is transferrin receptor, which has been identified as highly expressed and enriched in BECs by a number of data sets (Li et al., 2001; Enerson and Drewes, 2006), suggesting that other targets may also be identified. Interestingly, a peptide targeting Lrp1 has been shown to target molecules across the BBB, however, several genomic studies identified Lrp8, and not Lrp1, as highly expressed and enriched in BECs (Daneman et al., 2010a; Tam et al., 2012). For a detailed review on the expression of transporters at the blood-brain and blood-CSF interfaces in the developing and adult brain, see Saunders et al. (2013).

## 5. Emerging frontiers

### 5.1. Proteomics

One major criticism of transcriptomic approaches is that transcript abundance does not necessarily correlate with protein levels and actual functional activity. To that end, proteomic approaches may nicely complement gene expression studies. For a detailed review of proteomic studies which describe how various cell culturing conditions can significantly affect protein profiles and also how protein expression is explored using *in vivo* models of ischemia, stroke, inflammation, and disease, see Pottiez et al. (2009). Additionally, global genomic approaches are often followed with validation by qPCR assays and new developments in microfluidic devices have enabled higher throughput of expression validation studies from the same profiled samples, as well as the inclusion of a multitude of other samples, with or without experimental treatments, or from disease models and patients.

However, a major consideration of current proteomics BBB data is again the purity of the cell population being queried. Many BBB proteomics data are largely obtained from brain microvessel preparations consisting of additional pericytic or astrocytic connections that are in direct contact with BECs which are known to contain concentrated levels of cell-type specific receptors or channel proteins (see Table 2). Detection of known proteins such as those expressed on astrocytic endfeet (Chun et al., 2011), can be easily separated, however, lesser known hits may not be readily segregated by cell type or fall within a general category. A recent effort to profile one specific branch of CNS vasculature, the arteries comprising the Circle of Willis, generated an extensive data set of 6630 proteins, of which a third are specific to the cerebral arterial landscape (Badhwar et al., 2014). Interestingly, because isolation of the sampled tissue was via manual dissection and performed under light microscopy, whole units of arterial mediators such as neuronal regulators of vasoactivity, presumably from nerve terminals, as well as regulators of smooth muscle relaxation and contractility were identified. The cerebral artery-specific proteome was compared to the mouse microvessel proteome which revealed that intracortical microvessels had more BBB-unique protein numbers and that Circle of Willis arteries were less developed and specialized compared to microvessels (Badhwar et al., 2014). Additional studies have also compared human brain microvessel proteomic signatures with mouse (Uchida et al., 2011).

**Table 2:** **Recent proteomics data sets for the BBB**.

**Method of BBB isolation**	**Relevant study details**	**Species**	^*^Dev study	**Other tissues**	**Disease**
**LCM**					
Lu et al., 2008	Microvessels from CD31+ immunostained brain	Mouse			
Haqqani et al., 2005	*In vivo* ischemia	Rat			+
**FILTRATION/CENTRIFUGATION**					
Badhwar et al., 2014	Surgical dissection of circle of Willis brain artery	Mouse			
Ouyang et al., 2014	Mouse model of diet-induced obesity and control	Mouse			+
Searcy et al., 2014	Effect of aging on BMVs from wild type and Tg-SwDI mice	Mouse	+		+
Agarwal et al., 2012	BMVs from wild type and P-gp/Bcrp knockout	Mouse			
Chun et al., 2011	Membrane fraction of BMVs	Mouse			
Ito et al., 2011	BMVs from neonate, child, and adult	Cyno monkey	+		
Shawahna et al., 2011	BMVs from epilepsy patients and glioma patients	Human			+
Uchida et al., 2011	Human and mouse BMVs	Human and mouse			
Kamiie et al., 2008	Membrane fraction of microvessels from brain, liver and kidney	Mouse		+	
**CELL CULTURE**					
Ohtsuki et al., 2013	hCMEC/D3 compared to isolated human BMVs and HUVECs	Human		+	
Pottiez et al., 2010	BCECs co-cultured with glial cells	Cow			
Pottiez et al., 2009	BMECs cultured with and without astrocytes	Cow			
Lu et al., 2007	MECs from brain and heart	Rat		+	
Haqqani et al., 2007	Immortalized BECs and *in vitro* ischemia	Rat			+

* *Developmental stages. BCEC, brain capillary endothelial cell; BEC, brain endothelial cell; BMEC, brain microvessel endothelial cell; BMV, brain microvessel; hCMEC/D3, human cerebral microvascular endothelial cell line; HUVEC, human umbilical vein endothelial cell*.

Investigators have tried to circumvent these issues by coupling gene expression with proteomics in analyses of human brain vasculature isolated from resected glioma or epileptic lesions (Dauchy et al., 2008; Shawahna et al., 2011). Furthermore, a proteomics report of young and aged primate brain microvasculature provides a highly quantitative snapshot of membrane proteins expressed at the BBB at two stages of development (Ito et al., 2011). The data were compared to mouse BBB proteomic findings, which revealed significant differences. These comparisons may provide a cautionary note when translating findings between rodents and primates, likely impacting nervous system drug development studies focused on using mouse BBB models to understand transporter and receptor expression levels (Kamiie et al., 2008; Ito et al., 2011).

Overall, proteomic studies have been incredibly useful in identifying novel molecules. In particular, proteomic analysis of lipid rafts isolated from cultured cells has identified leukocyte adhesion molecules, ninjurin, Alcam, and Mcam, that are critical for trafficking of specific subsets of immune cells into the CNS during neuroinflammatory disease (Wosik et al., 2007; Cayrol et al., 2008; Ifergan et al., 2011). This suggests that the cultured cells may provide a model for “activated” endothelial cells and thus a good model to study their interaction with immune cells that might occur during neurological disease. In addition to these established functions of the BBB, these data sets may identify novel and unknown genes that could point to novel functions of the BBB. Although a daunting task, examining the function and regulation of each of these genes and pathways should provide incredible insight into how our brain interacts with the humoral space.

### 5.2. MicroRNA

MicroRNA (miRNA) arrays have also been used to identify and understand their role in regulating gene expression levels at the BBB in disease. Using hCMEC/D3 cells treated with TNFα and IFNγ, Reijerkerk et al. (2013) set out to profile the micro-RNAs that modulate BBB function under inflammatory conditions. With a panel of miRNAs identified to have differential expression induced by inflammatory mediators that also displayed opposing changes after barrier induction in the cell line, the authors further examined their expression in brain capillaries isolated via filtration from resected multiple sclerosis patient lesions. These samples showed decreased expression of miR-125a-5p (Reijerkerk et al., 2013). In another study using a miRNA array of 318 candidates, mouse brain endothelioma line bEND3 cells were profiled for altered miRNA expression in response to lupus serum or C5a (Eadon et al., 2014). Studies similar to these, for example evaluating freshly isolated BECs for miRNA expression at different developmental times or in diverse disease models, will further expand our knowledge of how gene expression is regulated at the BBB.

### 5.3. RNA-sequencing

RNA sequencing is a high-throughput, quantitative transcriptomic approach that is rapidly gaining popularity (Lister et al., 2008; Mortazavi et al., 2008; Nagalakshmi et al., 2008; Wilhelm et al., 2008), especially as more genome annotations are completed for more and more species. RNA-seq data also allows for the relatively unbiased discovery of alternative splicing within transcripts. Where the bulk of our transcriptome based knowledge has been driven by methods that illuminate overall gene expression (including microarray and SAGE), we have until recently, been lacking much of the important resolution afforded by identification of alternative splicing events. Many of the genes encoding proteins that form tight junctions and ABC transporters, for example, have distinct isoforms resulting from alternative splicing events. Future additional RNA-seq experiments with BBB data will provide a foundation for our future understanding of the relevance of such splicing behavior with respect to barrier development, maintenance, and dysfunction.

The high-throughput sequencing of transcriptomes afforded by RNA-seq technologies also facilitates the study of RNA editing. The process of RNA editing, which is most prevalent in the brain, is a post-transcriptional modification that can influence splicing patterns, coding sequence, and a host of other gene regulatory functions. Indeed, some of the best-studied RNA-editing events that result in altered protein isoforms occur within neuronal glutamate receptors. Future RNA-seq experiments will be needed to explore the role of RNA-editing within brain endothelial cells specifically.

Currently, BBB RNA-seq data is limited to a single dataset from mouse (at the time this review was submitted), which is already providing an extensive view of the expression landscape of not only BECs, but also neurons, astrocytes, oligodendrocytes, and microglia (Zhang et al., 2014). The authors of this study primarily used FACs and immunopanning methods to purify specific cell types, thus minimizing cross contamination and enabling greater quantitative comparisons. In addition, this valuable dataset has been made available to the public via a searchable website which includes differential splicing data (Zhang et al., 2014).

To supplement the paucity of RNA-seq data derived from the BBB, a recent developmental study using RNA sequencing to understand brain-CSF barrier formation assembled a comprehensive list of transcripts differentially enriched between E15 embryo and P42 adult rat lateral ventricle choroid plexus (Liddelow et al., 2013). This served as a quantitative approach to update a prior microarray study (Liddelow et al., 2012). The authors performed immunohistochemistry to demonstrate embryonic and choroid plexus epithelium-specific expression of genes they found enriched via RNA-seq (Liddelow et al., 2013).

The RNA-seq approach has also been used to profile expression signatures of single cells. In a study where cultured cortical neuron transcriptomes of single cells were compared to individual layer 5 pyramidal neurons *in situ*, the authors found that gene expression levels correlated better amongst single cultured neurons compared to the single neurons sampled *in situ* (Qiu et al., 2012). This *in vivo* heterogeneity may also apply to cells of the BBB and thus future gene expression studies sampling single BECs or capillary fragments from distinct disease regions or developmental stages of individual vascular branches may reveal further complexity in expression of specific transporters or receptors.

### 5.4. Final remarks

Advancements in techniques for the isolation and purification of BECs and in the technologies to study the resulting transcriptomes and proteomes make for an exciting and promising future for the BBB research community. Transcriptome wide data for human diseases associated with BBB dysfunction, and corresponding animal models are under-represented in the current selection of BBB data sets. Future studies may also identify sexual dimorphisms to the BBB, how the BBB changes in response to neuronal activity, during normal aging, as well as following intake of different drugs. Furthermore, understanding heterogeneity of the BBB between different segments of the vascular tree as well as between different regions of the brain may be important in our understanding of how the BBB interacts with neural cells to regulate brain function and behavior. Filling these gaps with data that are as close to the *in vivo* setting as possible, and utilizing techniques such as FACS or LCM, will be of great benefit. Finally, the continued commitment to share newly generated data sets in publicly accessible repositories will undoubtedly propel forward our understanding of BBB biology at an unprecedented pace.

### Conflict of interest statement

The authors declare that the research was conducted in the absence of any commercial or financial relationships that could be construed as a potential conflict of interest.

## Abbreviations

ABC: ATP binding cassette
BBB: blood-brain barrier
BEC: brain endothelial cell
FACS: fluorescence activated cell sorting
HBMEC: human brain microvessel endothelial cell
hCMEC/D3: human cerebral microvascular endothelial cell line
HUVEC: human umbilical vein endothelial cell
LCM: laser capture microdissection
miRNA: microRNA.
